# Does abolishing user fees for family planning increase contraception use? An impact evaluation of the national policy in Burkina Faso

**DOI:** 10.7189/jogh.12.04086

**Published:** 2022-10-14

**Authors:** Cheick Oumar Tiendrebeogo, Vena Joseph, Frank Bicaba, Alice Bila, Abel Bicaba, Thomas Druetz

**Affiliations:** 1 Department of Social and Preventive Medicine, School of Public Health, University of Montreal, Montreal, Quebec, Canada; 2 Société d’Études et de Recherches en Santé Publique, Ouagadougou, Burkina Faso; 3 Sciences de la Vie et de la Santé, University Aix-Marseille, Marseille, France; 4 Centre de recherche en santé publique, Montreal, Quebec, Canada; 5 Department of Tropical Medicine, Tulane University School of Public Health and Tropical Medicine, New Orleans, Louisiana, USA

## Abstract

**Background:**

Unmet needs for contraception constitute a major public health problem in sub-Saharan Africa. Several mechanisms have been tested to reduce the financial barrier and facilitate access to family planning services, with inconclusive results. Based on the positive impacts following the introduction of free health care for pregnant women, Burkina Faso decided to extend its national policy and abolished direct payment for family planning services. This study aims to evaluate the impact of this policy on contraceptive use and unmet needs for contraception among women of reproductive age (WRA) in Burkina Faso.

**Methods:**

This study uses two different study designs to examine the impact of a user fee removal policy on contraceptive use across a panel of 1400 households randomly selected across eight health districts. Data were collected using a standardized socio-demographic questionnaire at three different time points during the pilot and scale-up phases of the fee abolition program. The questionnaire was administered six months after the launch of the pilot fee abolition program in four health districts. For the remaining four health districts, the survey was conducted one year prior to and six months after the implementation of the program in those areas. All WRA in the households were eligible to participate. A cross-sectional study design was used to determine the association between knowledge of the fee abolition policy among WRA and actual use of contraceptives by WRA six months after the policy’s implementation and across all eight districts. Additionally, a pre-post study with a non-randomized, reflexive control group was designed using repeated surveys in four health districts. Hierarchical logistic mixed effects models were adjusted for a set of time-variant individual variables; the impact was assessed by a difference-in-differences approach that compared pre-post changes in contraception use in women who knew about the new policy and those who did not.

**Results:**

Of the 1471 WRA surveyed six months after the removal of user fees for family planning services, 56% were aware of the policy’s existence. Knowledge of the fee abolition policy was associated with a 46% increase probability of contraceptive use among WRA six months after the policy’s implementation. Among the subset of the participants who were surveyed twice (n = 507), 65% knew about the fee removal policy six months after its introduction and constitute the intervention group. Pre-post changes in contraceptive use differed significantly between the intervention (n = 327) and control groups (n = 180). Removing user fees for family planning led to an 86% (95% confidence interval (CI) = 0.49, 1.31) increase in the likelihood of using contraception. In the study area, the policy reduced the prevalence of unmet needs for contraception by 13 percentage points.

**Conclusions:**

Removing user fees for family planning services is a promising strategy to increase access to, and reduce unmet needs for, contraception. A broader dissemination of the policy’s existence will likely increase its impact on the overall population.

In line with the 1986 Bamako Initiative and the structural adjustment programs enforced in the 1980s and 1990s, many low- and middle-income countries (LMICs) introduced user fees for health services, including for contraception and family planning [1]. These fees, in the form of direct payments, provided additional revenue sources to finance the countries’ health systems. The rationale was that this income would help ensure the health facilities’ operational and financial autonomy and improve the quality of their services. Although the fees may have contributed to the sustainability of the health care services, there is evidence that higher costs of family planning services are associated with a reduction in contraception use [2-4]. User fees represent an obstacle to these services, especially among the poorest women living in rural areas [5,6].

Lack of access to family planning services constitutes a major public health problem in sub-Saharan Africa (SSA) [7,8]. With an average of 4.7 children per woman, the fertility rate in SSA remains the highest in the world [9]. The proportion of women of reproductive age (WRA) with unmet needs for contraception (UNC) currently stands at 25% in the continent, comprising ~ 47 million women. The effects of these unmet needs represent a devastating public health crisis by contributing to the 19 million unsafe abortions performed annually in SSA, a leading cause of maternal death [10]. UNC are also associated with an increased risk of contracting sexually transmitted diseases, ultimately leading to higher morbidity and mortality in children and women [11-15]. Failure to provide women with adequate access to contraception violates their rights to sexual and reproductive health and constitutes a key issue of gender inequity [16,17].

To overcome financial barriers and allow women access to contraceptive methods, several mechanisms have been tested (such as cash transfer programs, distribution of free vouchers, and community-based or performance-based financing) with inconclusive results [7,18,19]. Another type of intervention relied on demand elasticity and aimed to increase access to family planning services by subsidizing prices or alleviating user fees. Again, the evidence is scarce and inconclusive [20]. While a study in Colombia showed that a reduction in the price of levonorgestrel-releasing implants was immediately accompanied by an increase in demand, studies in Indonesia and Bangladesh showed that sales of different types of contraceptives were inelastic to price reductions [21-23]. Recent evidence gathered from eight sub-Saharan countries has suggested that rendering family planning services completely free of charge might be a promising strategy to increase access to contraception [24]; however, several systematic reviews have found no rigorous evaluations of the effects of user fee removal initiatives on contraception use [20,25].

The opportunity to provide such evidence presented itself in Burkina Faso. Although user fees for pregnant women and children under five were removed in 2016, the country decided three years later to extend the free health care policy to include family planning services. As a first stage, the extension was piloted in two regions, Cascades and Centre-West, where family planning services became free of charge in July 2019. A year later, the pilot was scaled up nationally. Despite supply shortages, insufficient communication, and other factors that limited the policy’s coverage [26], its implementation was deemed successful, but further evidence is needed to determine whether the new policy has affected contraceptive use among women. We designed a pre-post study with a non-randomized control group to assess the effects of the user fee removal policy on contraceptive use in women of reproductive age (WRA) in Burkina Faso.

## METHODS

### Abolition of user fee for family planning services

In 2016, Burkina Faso introduced a national policy that abolished user fees for women and children at public health facilities. The policy originally covered most services related to pregnancy and reproductive health, but did not include family planning (FP) services, for which costs remained in place. Three years later, in July 2019, a pilot program in two regions of Burkina Faso (Centre-West and Cascades) expanded the no-fee policy to include FP, and a national scale-up of the extension was implemented in July 2020.

The new policy covers 100% of the cost of FP consultations, counselling, tests, examinations, and contraceptives themselves (injectables, implants, copper intrauterine devices, emergency contraceptive pills, condoms, surgical methods, and a range of natural methods). More information about the policy’s implementation is available elsewhere [26].

### Study design

**Figure 1:**
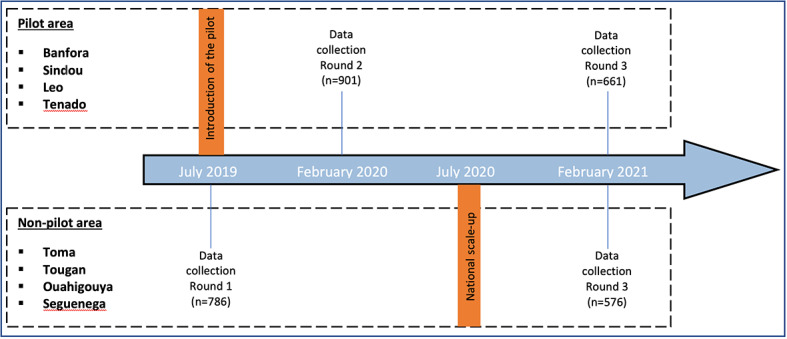
Timeline of the policy introduction and the surveys.

The study used data from three rounds of surveys that were originally planned for another study (SYNERGIE) and were conducted in July 2019 (round 1), February 2020 (round 2), and February 2021 (round 3). Rounds 1 and 3 surveyed the same set of households in four health districts that were not part of the pilot, while round 2 surveyed households in four health districts located in the pilot area, as presented in **Figure 1**. The three rounds followed the same sampling and survey procedures.

This platform allowed the research question to be explored through two different study designs. The first is a cross-sectional assessment of the knowledge of the policy’s existence among the targeted population and its association with the use of contraceptives by WRA, conducted six months after the policy was introduced. Data from rounds 2 and 3 were used for this analysis. The second is a pre-post design with a non-randomized control group (ie, a quasi-experimental design) applied to evaluate the policy’s effects on contraceptive use among WRA. This longitudinal design used data from rounds 1 and 3, which took place one year before and six months after the introduction of the new policy, respectively. This was a natural experiment; the study was not initially designed to assess the new policy’s effects.

### Sampling

The sampling procedures were derived from those of the Demographic and Health Surveys program. A two-stage cluster sampling was carried out in eight health districts: Leo, Tenado, Sindou, and Banfora in the pilot area, and Toma, Tougan, Ouahigouya, and Seguenega outside the pilot area. These districts were purposively selected based on two criteria: they covered mostly rural areas and they were not located in regions considered to be red (ie, areas that are affected by frequent terrorist attacks).

In each district, four to eight enumeration areas were randomly selected with a probability proportional to the size of their population. In the second stage, 24 households per area were randomly selected with equal probability. The maximum target size per round was 700 households, which was set for the purpose of another study on the effects of removing user fee in health facilities for all children under five [27,28].

Only households with ≥1 woman aged 15-49 were eligible. Ineligible households and households that could not be found were replaced by the nearest one, but only at round 2. For logistical reasons, no replacement was carried out during round 1. Round 3 was a follow-up survey administered to the households that had already participated during round 1; losses to follow-up were not replaced.

All women aged 15-49 years, living in the sampled households and present at the time of the survey were eligible and invited to participate in the study during the door-to-door survey. A flowchart of the recruitment process by year and type of area (pilot vs non-pilot) is provided in Figure S1 in the **Online Supplementary Document**.

### Survey procedures

A standardized sociodemographic questionnaire was administered individually to all participants. Questions were extracted from the Demographic and Health Surveys instruments. The surveys gathered data on household features and the participants’ main sociodemographic characteristics. A module of the questionnaire specifically targeted reproductive health indicators – notably, the participants’ knowledge about the existence of the new policy, their current use of contraceptives, their sexual activity, their childbearing intentions and pregnancy history, and whether they were recently in contact with a health professional.

Surveys were individually administered at the participants’ homes by female research assistants with relevant experience and training in survey administration. Most female research assistants (>80%) were hired for more than one survey round. The survey was translated into the local languages, and participants answered in the language of their choice (Moore, Dioula, Lélé, Fufuldé, Gourmantche). Responses were collected electronically on tablets using Commcare software (Dimagi, Cambridge, USA). Data were automatically uploaded to a secure server.

### Analyses

The primary outcome under study was the use of contraception by WRA. Its association with knowledge about the policy’s existence was explored cross-sectionally in a multilevel logistic model with random intercepts at the household, commune, and district levels. Potential confounding variables were identified by reviewing the literature [29-31] and were tested in the models: age, number of children, occupation, education level, being sexually active, matrimonial status, visit to a health facility in the last three months, childbearing preferences, previous experience of miscarriage/abortion, having received a household visit from a health care worker to discuss family planning, and type of setting (rural/urban). Descriptive analysis using Pearson χ2 tests was performed to assess differences in these characteristics among WRA according to their knowledge of the policy. A backward stepwise model selection process was done by examining the Akaike information criterion values. The model with the lowest AIC value and the models within two AIC units of that value were examined; the most parsimonious among them was selected. The final model was replicated on the secondary outcome under study (ie, having UNC) which includes: 1) WRA who are sexually active, are not using any method of contraception, and report not wanting any more children or wanting to delay the next pregnancy, and 2) WRA with an unwanted pregnancy [32].

Longitudinal analyses were restricted to the participants in the four districts who were surveyed twice. WRA who self-reported knowing about the policy in the 2021 survey constituted the exposure group; WRA who self-reported not knowing about the new policy constituted the control group. Fixed effects were included in the logistic models to isolate changes only attributable to time-varying factors within individuals. Only individual time-varying variables among the list of potential confounders were tested in the model [33]. Based on the conclusive result of the Hausman test and efficiency considerations, a hierarchical mixed effects model was fitted, with results presented below (results from fixed effects models are presented in Document S4 in the **Online Supplementary Document**) [34]. Effects were assessed following a difference-in-differences approach, which allows controlling for observed and unobserved time-invariant confounders [35]. Pre-post changes in contraceptive use were compared between the exposure and control groups by adding an interaction term between period and exposure. Missing data represented less than 1% of the observations and were excluded from the analyses. Losses to follow-up were excluded from the longitudinal analyses since there was no statistical difference according to contraceptive use status.

Risks were derived from logistic regression models by computing marginal standardized probabilities and using the margins and nlcom Stata commands [36]. All analyses were performed using Stata version 14.0 software (StataCorp LLC, College Station, Texas) and robust covariance estimators were consistently used [37].

### Ethics

All participants recruited in 2019 and 2020 provided written informed consent. As suggested and approved by the research ethics committees, all participants recruited in 2021 also provided informed consent verbally to reduce risk of COVID-19 transmission. The questionnaire was administered individually in a secluded area to preserve participant confidentiality. Participants aged 15-17 years old were considered mature minors and consented as adults, as per national standards. All study procedures, including those for obtaining consent, were approved by the Comité d’éthique de la recherche en sciences de la santé at University of Montreal (Certificate #CERSES-20-146-D) and the Comité d’éthique pour la Recherche en Santé in Burkina Faso (Deliberation #2018-6-075).

## RESULTS

A total of 1471 participants were surveyed six months after the removal of user fees for family planning services (**Table 1**). Among those, 818 (56%) knew about the existence of the new policy. When comparing the participants who knew about it and those who did not, there were significant differences in key sociodemographic characteristics, including age, marital status, primary occupation, sexual activity, number of children, and having recently visited a health facility or having been visited by a health professional. There were also significant variations according to the health district.

**Table 1:** Socio-demographic characteristics of the participants six months after the removal of user fees for family planning services in 8 health districts in Burkina Faso (n = 1471)

	Knows the existence of the new policy		
**Characteristics**	yes (%)	n (%)	*P*
**Participants**	818	653	NA
**Age group (year)**			
≤25	249 (0.31)	236 (0.37)	
26-35	295 (0.36)	191 (0.30)	0.013
>35	265 (0.33)	211 (0.33)	
**Married or in a relationship**	690 (0.84)	504 (0.77)	<0.001
Recently visited a health facility*	629 (0.77)	417 (0.64)	<0.001
**Childbearing preferences**			
Wants a/another child	584 (0.71)	466 (0.71)	
Does not want children (anymore)	140 (0.17)	112 (0.17)	0.994
Impossible to get pregnant	16 (0.02)	14 (0.02)	
Does not know	78 (0.09)	61 (0.09)	
**Being sexually active**	758 (0.93)	559 (0.85)	<0.001
**Number of children**			
0	151 (0.18)	156 (0.24)	
1-2	182 (0.22)	125 (0.19)	0.017
3-4	216 (0.26)	144 (0.22)	
≥5	268 (0.33)	228 (0.35)	
**Went to primary school**	343 (0.42)	267 (0.41)	0.726
**Household size**			
1-5	226 (0.27)	154 (0.23)	
6-10	399 (0.49)	314 (0.48)	0.070
≥11	193 (0.23)	184 (0.28)	
**Primary occupation**			
Commerce	276 (0.34)	180 (0.27)	
Agriculture	30 (0.03)	14 (0.02)	0.001
Housekeeping	448 (0.55)	379 (0.58)	
Other	63 (0.07)	79 (0.12)	
**Receives monetary retribution for her work**	355 (0.43)	261 (0.40)	0.203
Had recently a miscarriage, abortion, or stillbirth*	160 (0.19)	124 (0.19)	0.641
**Uses contraception**	331 (0.40)	182 (0.28)	<0.001
**Health district**			
Toma	61 (0.07)	60 (0.09)	
Tougan	79 (0.09)	20 (0.03)	
Banfora	242 (0.29)	269 (0.41)	
Sindou	31 (0.04)	89 (0.13)	<0.001
Tenado	51 (0.06)	19 (0.03)	
Leo	129 (0.16)	69 (0.10)	
Ouahigouya	164 (0.20)	74 (0.11)	
Seguenega	61 (0.07)	53 (0.08)	
**Household is located in an urban area**	383 (0.47)	289 (0.44)	0.274
Household was recently visited by a health professional to speak about family planning*	138 (0.17)	63 (0.10)	<0.001

NA – not applicable

*In the last 12 mo.

### Contraceptive use six months after the policy’s introduction

**Figure 2:**
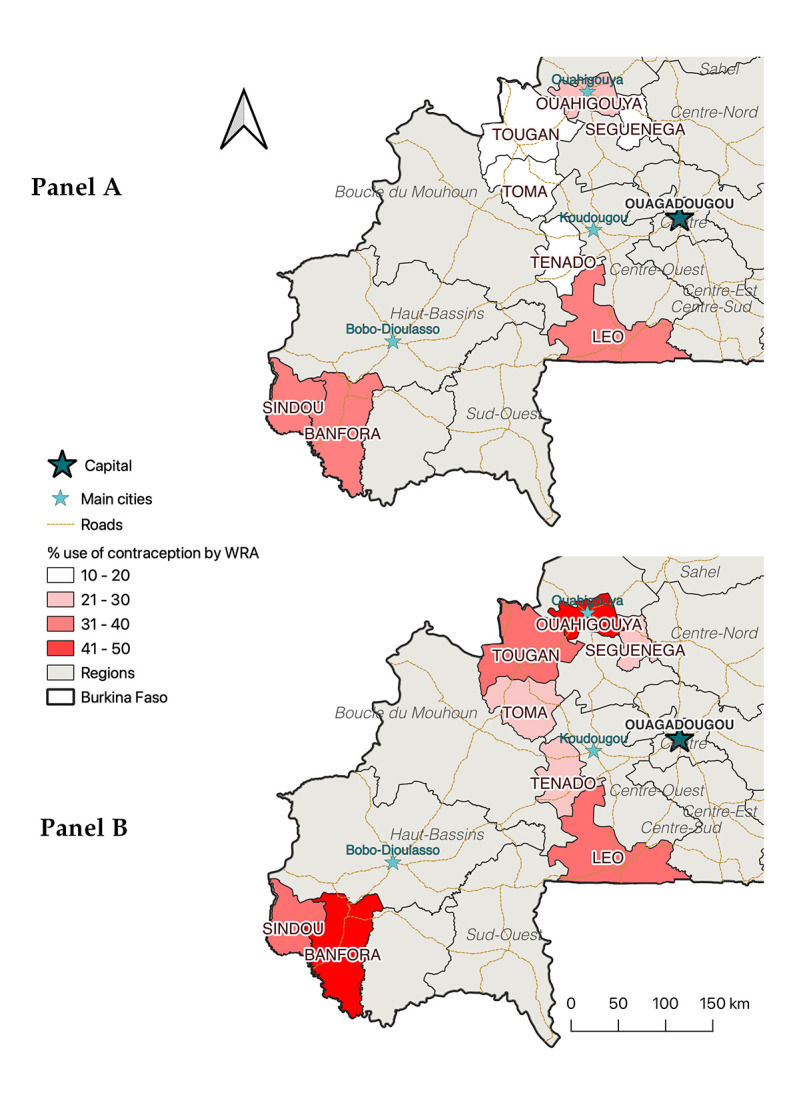
Prevalence of contraceptive use, six months after removing user fee for family planning services, stratified by district and according to the policy knowledge of the participants. Panel A shows prevalence among participants who did not know about the policy; Panel B shows prevalence among participants who knew about the policy.

**Figure 2** shows the crude prevalence of contraceptive use at the district level, six months after the introduction of the new policy, stratified according to the policy knowledge of the participants. In each of the eight districts under study, prevalence was higher among those whose knew about the new policy compared to those who did not. Multiple regression analysis confirms that the adjusted odds of using contraception were 1.94 times higher among those who knew about the policy compared to those who did not; the outputs are presented in Document S2 in the **Online Supplementary Document**. Six months after its introduction, knowing about the new policy was therefore significantly associated with a 46% (95% confidence interval (CI) = 23%, 74%) increased probability of using contraception among WRA (**Table 2**).

**Table 2:** Association between knowing about the user fee abolition policy and contraception use among women of reproductive age, six months after the policy’s introduction*

Knowing about the fee abolition policy	Predicted probability of using contraception	95% CI	Risk ratio	95% CI
Yes (n = 828)	0.375	0.342, 0.409	1.466	1.236, 1.738
No (n = 653)	0.256	0.231, 0.281		

CI – confidence interval

*The association was estimated six months after the introduction of the new policy in eights health districts (N = 1471). The multilevel logistic model was fitted with random intercepts at the household and district levels, and was adjusted for age, number of children, receiving a salary, having recently visited a health center, being sexually active, and type of setting.

**Figure 3:**
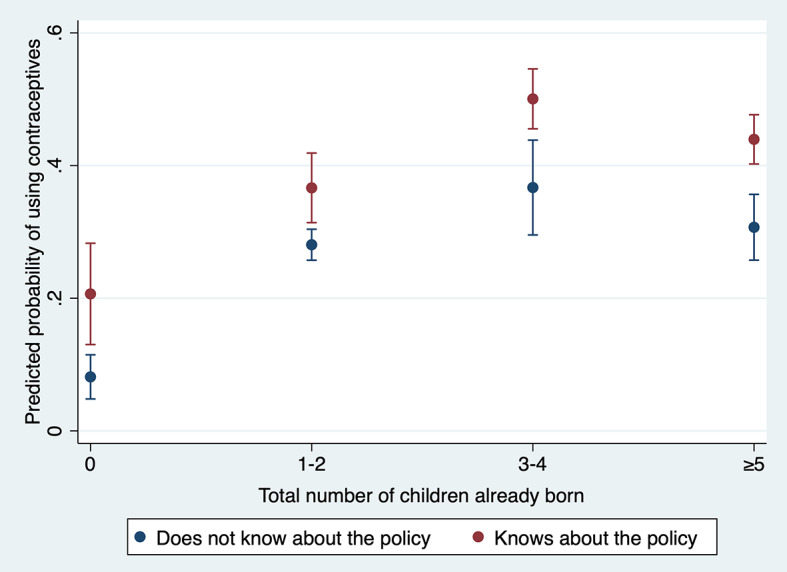
Predicted contraception use among women of reproductive age six months after the introduction of user fee abolition, by knowledge of the policy and number of children already born.

The odds of contraceptive use were also significantly lower in older WRA and among those living in rural areas; odds were significantly higher in multiparous WRA, those who had recently visited a health facility, and those who were remunerated at their work; the results are fully presented in Document S2 in the **Online Supplementary Document****.** **Figure 3** shows the effect of knowing about the policy on contraceptive use according to the number of children already born.

### Effect of user fee removal on contraceptive use

**Figure 4:**
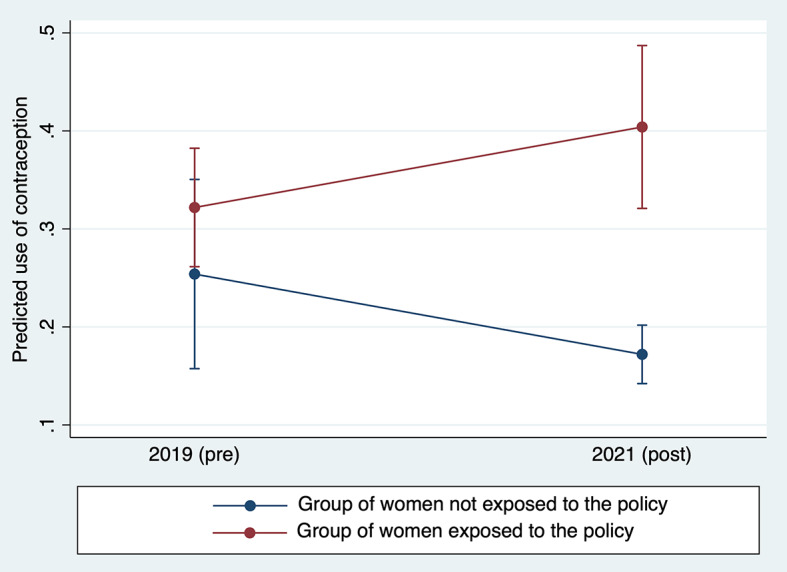
Mean contraceptive use among women of reproductive age, before and after the introduction of the policy, in the groups of women exposed and non-exposed to the policy.

In four districts, the WRA were surveyed twice: first in July 2019, one year before the new policy, and a second time in January 2021, six months after the removal of user fees. Of the 786 WRA surveyed at baseline in 2019 (round 1), 279 were lost to follow-up in 2021, for an overall response-rate of 65% in the pre-post study; the data are presented in Table S3 in the **Online Supplementary Document**. In the sub-sample of 507 WRA surveyed twice, knowledge of the policy’s existence was prevalent in 65% (n = 327) six months after the policy’s implementation. Pre-post changes in contraceptive use differed significantly between the group of WRA who would later indicate familiarity with the policy in the 2021 survey (intervention group) vs the group of WRA who would not (**Figure 4**). The ratio of risk ratios indicates that removing user fees effectively increased the likelihood of using contraception by 86% (95% CI = 49%, 131%).

The Hausman test does not indicate that a model with fixed effects at the individual level should be preferred over a model with random effects (Document S4 in the **Online Supplementary Document**). When using fixed effects, the coefficient of the effects estimator is slightly reduced, but remains statistically significant (ratio of risk ratios = 1.52, 95% CI = 1.15, -2.02).

### Effect of user fee removal on unmet needs for contraception

The proportion of women with UNC in 2019 was 41% in the control group and 34% in the intervention group (the group of women who knew about the user fee policy in 2021). At baseline, the difference between the two groups was not statistically different; however, while UNC increased to 50% in the control group, it decreased to 30% in the intervention group, which translates into an absolute prevalence reduction of 13 percentage points (**Table 3**). The ratio of risk ratios indicates that the user fee removal policy decreased the individual likelihood of presenting UNC by 27% (95% CI = 0.11, 0.41).

**Table 3:** Absolute and relative effects of the user fee removal policy on unmet need for contraception in four districts of Burkina Faso*

	Prevalence of UNC		Absolute effects		Relative effects	
	Pre-intervention	Post-intervention	Difference in risk differences	95% CI	Ratio of risk ratios	95% CI
**Control group**	0.42	0.50	-0.126	-0.236, -0.018	0.728	0.594, 0.892
**Intervention group**	0.34	0.30				

UNC – unmet need for contraception, CI – confidence interval

*Predicted probabilities were derived from a multilevel logistic model with robust variance estimators and random intercepts at the individual, household, and districts levels (N = 964). The model was adjusted for age, number of children, receiving a salary, and type of setting. Relative effects were estimated by the ratio of risk ratios (exposed group vs control group, 2019 vs 2021).

## DISCUSSION

This study shows that removing user fees for family planning services increases access to contraception among WRA in rural Burkina Faso and reduces UNC. While previous studies have shown a similar increase in demand for health care services after removing user fees for children <5 and for pregnant women [38-40], this is the first study to examine the effects of such a policy on the demand for family planning services.

Unfortunately, it is not possible to compare our results with the few evaluations of other types of interventions implemented to reduce the financial barrier to family planning services (such as free vouchers, performance-based financing, or cash transfer) since most of those studies were ecological or used a pre-post design without a control group [20]. However, with a demonstrated 86% increase in contraceptive use and 13 percentage point reduction in the prevalence of UNC, the abolition of user fees is arguably one of the most effective financial strategies for increasing access to family planning services in LMICs. It is expected that the population impact of the new policy will increase as a greater proportion of the population becomes aware of its existence: For reasons explored elsewhere, only 55% of the participants knew about the new policy six months after its introduction [26,41].

While previous studies have shown an upward trend in contraceptive use over the past ten years in Burkina Faso, our study shows no statistically significant change between 2019 and 2021 in the control group [42-44]. It is plausible that the COVID-19 pandemic has restricted (or slowed the improvement in) access to family planning services, as was observed in West Africa during the 2013-2016 Ebola crisis [45]. This hypothesis is consistent with a recent study in Burkina Faso showing that demand for contraception has grown faster than access during the COVID-19 pandemic, resulting in an increased proportion of WRA with unwanted pregnancies [27].

Finally, while removing user fees is critical to increasing access to family planning services, two important considerations need to be outlined. First, as suggested by the results, women who are younger, unmarried, not yet sexually active/without children, or not in recent contact with a health facility are less likely to know about the new policy. Arguably, these inequities will disappear as the policy becomes more entrenched, but since UNC is already higher among these vulnerable subgroups in Africa [46], sensitization campaigns targeting adolescents and young women should be implemented to avoid an increase in health inequities. Second, user fee abolition policies tend to increase women’s agency, but they are not sufficient to combat the multifactorial influences limiting women’s empowerment and gender equality [41,47]. Future interventions should tackle the non-financial barriers to reproductive health and focus on the promotion of gender-equitable access to health care.

### Strengths and limitations

The impact of fee removal was estimated in this study using one of the most robust evaluation designs for studies in which randomized assignation to an intervention is not possible, ie, a pre-post design with control group [48,49]. The hierarchical structure of the data was considered, with random intercepts at the household, commune, and district levels. Time-invariant observable and non-observable confounding factors were controlled for by following a difference-in-differences approach, and the influence of potential individual, time-variant confounding variables was tested in the models [50,51].

It was not possible to test the assumption that trends were similar between the exposed and control groups prior to the baseline, but it is reassuring that there was no significant difference in contraceptive use (nor in UNC) between the two groups at the baseline. The presence of reverse causality bias cannot be ruled out, as some women may have learned about the policy when they went to obtain a contraceptive method at the health facility. Unfortunately, the two temporalities (of knowledge and decision) were impossible to distinguish in this natural experiment, even by using fixed effects at the individual level [33].

## CONCLUSIONS

Burkina Faso is one of the first countries in sub-Saharan Africa to introduce a policy removing user fees for family planning services. We provide evidence that this policy is a promising strategy to increase access to and reduce unmet needs for contraception. The user fee removal led to an 86% increase in contraceptive use by WRA in rural Burkina Faso and reduced the prevalence of unmet need for contraception by 13 percentage points. However, these significant improvements in access and use of contraceptives were tempered by the limited awareness of the fee abolition policy among its target population, notably the adolescents and unmarried women. A broader dissemination of the policy’s existence will likely further contribute to improve the sexual and reproductive health of WRA in rural Burkina Faso.

## Additional material:


Online Supplementary Document
