# Supplementary material for: Does abolishing user fees for family planning increase contraception use? An impact evaluation of the national policy in Burkina Faso
Source: J Glob Health. 2022 Oct 14;12:04086. doi: 10.7189/jogh.12.04086 (PMC9559360; doi:10.7189/jogh.12.04086)
Supplement: Online Supplementary Document [file jogh-12-04086-s001.pdf]

Online Supplementary Document

Figure S1

A. Recruitment process, 2019, non-pilot area

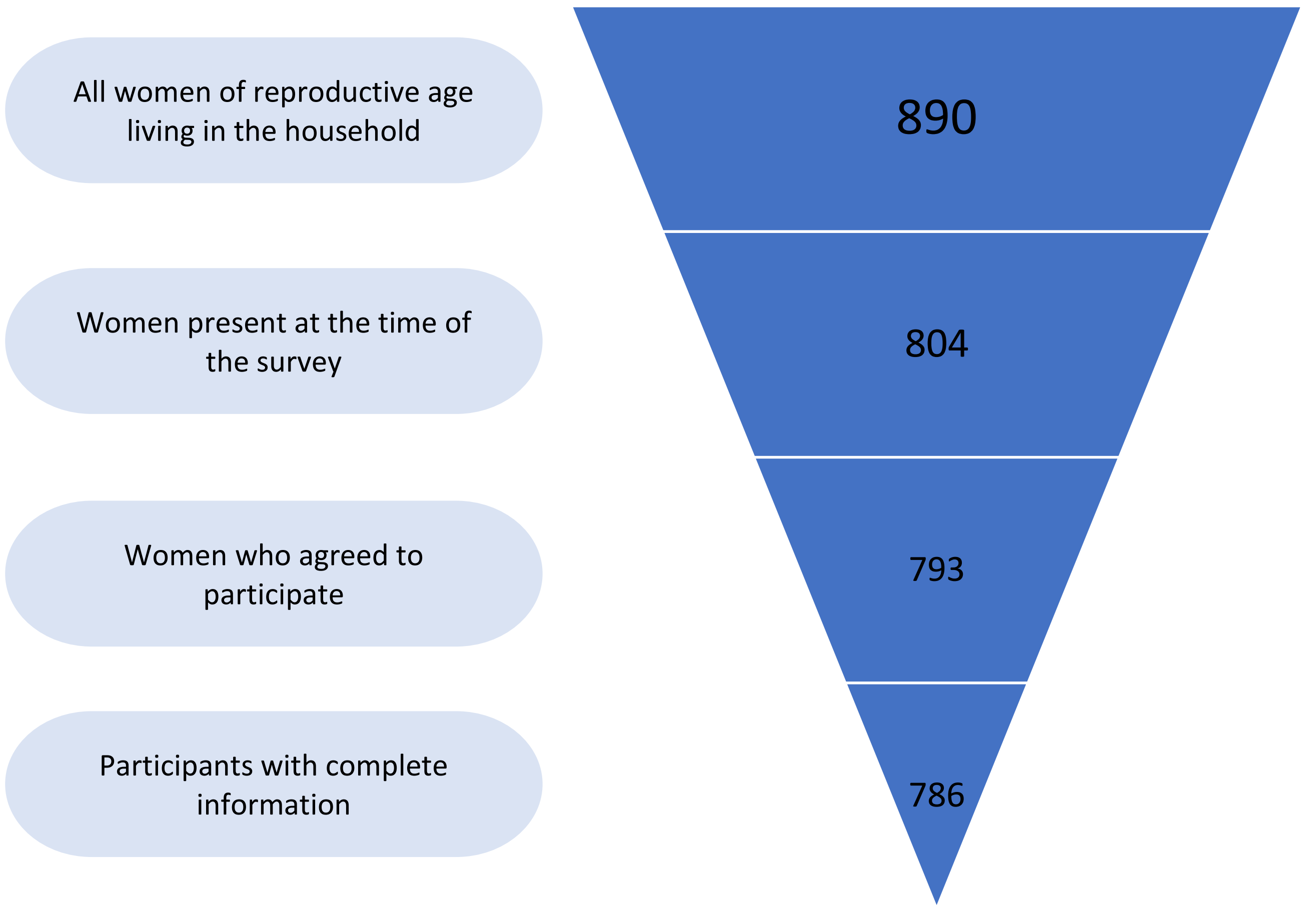

B. Recruitment process, 2020, pilot area

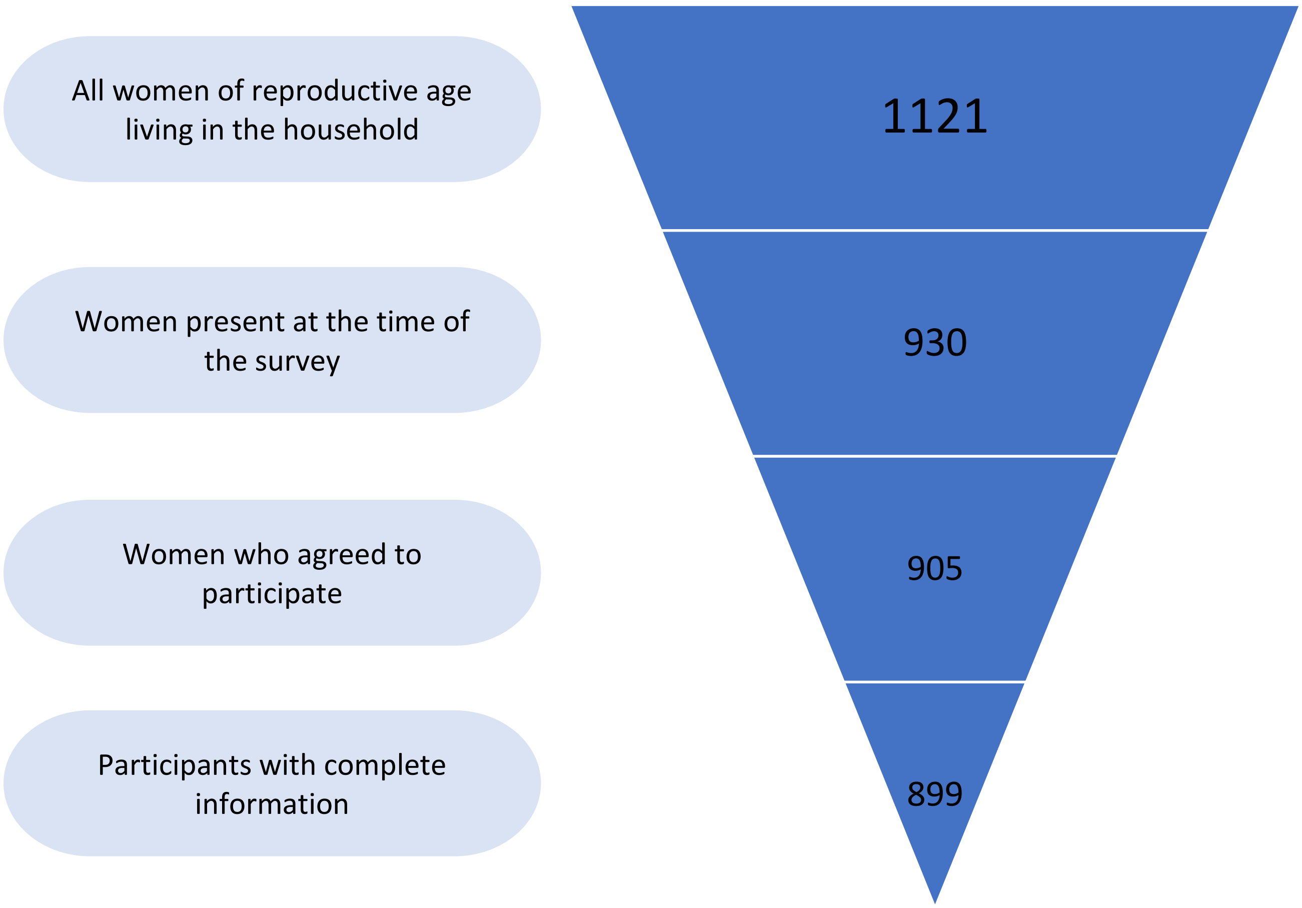

C. Recruitment process, 2021, non-pilot area

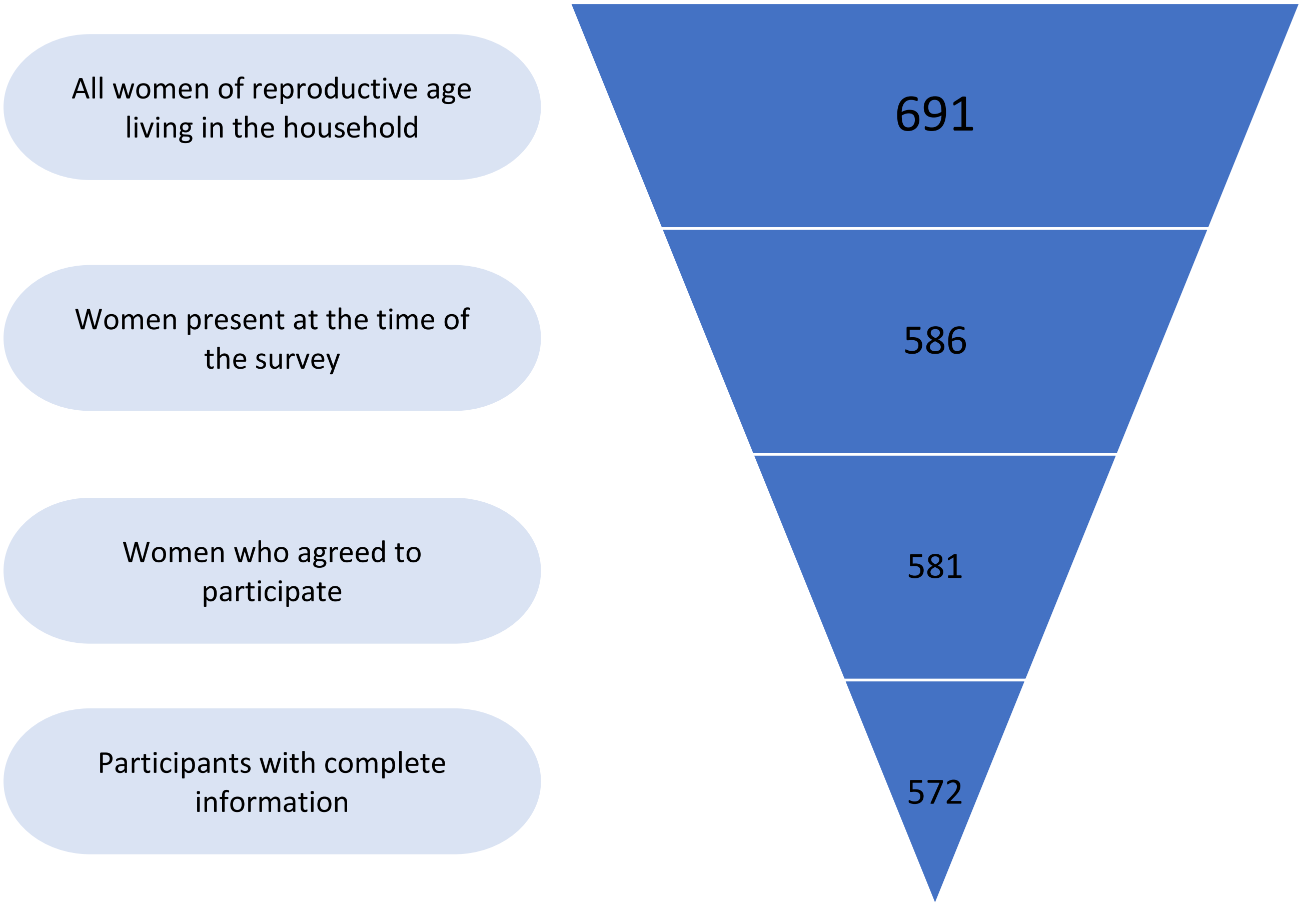

Document S2 Output of mixed effects logistic regression model with current use of contraception (yes/no) as dependent variable

All analyses were conducted using Stata.
More information about the commands and the results' interpretation is available at:
https://www.stata.com/manuals/memelogit.pdf

Mixed-effects logistic regression Number of obs = 1,431

Grouping information

Group variable	No. of groups	Observations per group: Minimum	Average	Maximum
district	8	70	178.9	510
commune	26	21	55.0	330
household	1,054	1	1.4	5

Integration method: mvaghermite Integration pts. = 7

Wald chi2(7) = .
Log pseudolikelihood = -823.4155 Prob > chi2 = .
(Std. err. adjusted for 8 clusters in ds)

Uses contraception	Odds ratio	Robust std. err.	z	P>|z|	[95% conf.	interval]
Knows the existence of the new policy	1.941799	.2871536	4.49	0.000	1.45321	2.594658
Age category (ref.: 16-24 years)						
26-35 years	.7082971	.158677	-1.54	0.124	.4565877	1.09877
>35 years	.5056738	.1430906	-2.41	0.016	.2904062	.8805117
Has recently visited the health facility	1.500877	.1150966	5.29	0.000	1.291426	1.744297
Number of children already born						
1-2	1.280997	.1721367	1.84	0.065	.9843873	1.666979
3-4	3.12849	1.232216	2.90	0.004	1.445682	6.770129
>4	3.491859	1.29911	3.36	0.001	1.684132	7.239982
Is gainfully employed	1.724735	.2538543	3.70	0.000	1.292524	2.301474
Lives in rural area	.5080609	.0784336	-4.39	0.000	.3754124	.6875794
Is sexually active	7.37504	3.291487	4.48	0.000	3.075193	17.68709
District						
Tougan	1.364038	.1246187	3.40	0.001	1.140409	1.63152
Banfora	2.074509	.238581	6.35	0.000	1.655854	2.599014
Sindou	2.041098	.1689495	8.62	0.000	1.735429	2.400607
Tenado	.9243087	.038549	-1.89	0.059	.8517596	1.003037
Leo	1.319591	.097847	3.74	0.000	1.141099	1.526004
Ouahigouya	1.92962	.1823125	6.96	0.000	1.603428	2.322171
Seguenega	.7574312	.0548373	-3.84	0.000	.6572295	.8729097
_constant	.0165966	.0065637	-10.36	0.000	.0076451	.0360295
district						
var(_cons)	2.97e-37	5.23e-36			2.93e-52	3.01e-22
district>commune						
var(_cons)	2.65e-36	7.96e-36			7.29e-39	9.61e-34
district>commune>household						
var(_cons)	1.010064	.3931223			.4710392	2.165913

Note: Estimates are transformed only in the first equation to odds ratios.
Note: _cons estimates baseline odds (conditional on zero random effects).

Table S3 Socio-demographic characteristics of the participants surveyed in 2019, according to their follow-up status in 2021

Characteristics	Lost during follow-up		
	yes (%)	no (%)	p-value
Participants	279	507	NA
Age group (year)			
≤25	124 (0.45)	131 (0.26)	
26-35	86 (0.31)	180 (0.36)	< 0.001
>35	61 (0.23)	184 (0.37)	
Married or in a relationship	219 (0.78)	455 (0.89)	< 0.001
Recently visited a health facility¶	167 (0.60)	336 (0.66)	0.083
Childbearing preferences			
Wants a/another child	177 (0.70)	295 (0.63)	
Does not want children (anymore)	47 (0.19)	111 (0.24)	0.211
Impossible to get pregnant	4 (0.02)	14 (0.03)	
Does not know	24 (0.10)	46 (0.10)	
Being sexually active	249 (0.89)	479 (0.95)	0.005
Number of children			
0	62 (0.22)	64 (0.12)	
1-2	92 (0.33)	99 (0.20)	< 0.001
3-4	56 (0.20)	129 (0.25)	
≥5	68 (0.24)	215 (0.42)	
Went to primary school	119 (0.43)	159 (0.31)	0.002
Household size			
1-5	31 (0.11)	65 (0.13)	
6-10	103 (0.37)	222 (0.44)	0.069
≥11	145 (0.52)	220 (0.43)	
Primary occupation			
Commerce	50 (0.18)	119 (0.23)	
Agriculture	74 (0.27)	96 (0.19)	0.114
Housekeeping	137 (0.49)	269 (0.53)	
Other	17 (0.06)	23 (0.05)	
Receives monetary retribution for her work	54 (0.23)	146 (0.29)	0.096
Had recently a miscarriage, abortion or stillbirth¶	48 (0.17)	123 (0.24)	0.033
Uses contraception	77 (0.28)	154 (0.31)	0.411
Health district			
Toma	32 (0.11)	110 (0.22)	
Tougan	42 (0.15)	86 (0.17)	0.001
Ouahigouya	149 (0.53)	216 (0.43)	
Seguenega	56 (0.20)	95 (0.19)	
Household is located in an urban area	117 (0.43)	179 (0.36)	0.051
Household was recently visited by a health professional to speak about family planning¶	100 (0.36)	179 (0.35)	0.883

¶ in the last 12 months

Document S4 Results from the Hausman test

All analyses were conducted using Stata.
More information about the commands and the results' interpretation is available at:
https://www.stata.com/manuals/xtxtlogit.pdf
https://www.stata.com/manuals/rhausman.pdf

Model with fixed effects

```
Conditional fixed-effects logistic regression        Number of obs    =    326
Group variable: id                                   Number of groups =    163

                                                     Obs per group:
                                                                  min =      2
                                                                  avg =    2.0
                                                                  max =      2

                                                     LR chi2(2)       =   9.44
Log likelihood = -108.26368                          Prob > chi2      = 0.0089

                                     ----------------------------------------------------------------------------
                                Uses contraception | Odds ratio   Std. err.      z    P>|z|     [95% conf. interval]
                                     -------------+--------------------------------------------------------------
            Knows the existence of the new policy |   2.607099   .9154788     2.73   0.006     1.309964    5.188665
                                                  |
                                Year (ref. = 2019) |
                                             2021  |   .6333333   .1856919    -1.56   0.119      .356502     1.12513
                                     ----------------------------------------------------------------------------
```

Model with random effects

```
Random-effects logistic regression                   Number of obs    =  1,007
Group variable: id                                   Number of groups =    507

Random effects u_i ~ Gaussian                        Obs per group:
                                                                  min =      1
                                                                  avg =    2.0
                                                                  max =      2

Integration method: mvaghermite                      Integration pts. =     12

                                                     Wald chi2(2)     =  24.64
Log likelihood = -604.27359                          Prob > chi2      = 0.0000

                                     ----------------------------------------------------------------------------
                                Uses contraception | Odds ratio   Std. err.      z    P>|z|     [95% conf. interval]
                                     -------------+--------------------------------------------------------------
            Knows the existence of the new policy |   3.815726   1.081318     4.73   0.000     2.189575    6.649587
                                                  |
                                Year (ref. = 2019) |
                                             2021  |   .4925898   .1261665    -2.76   0.006     .2981729    .8137718
                                                  |
                                         _constant |   .3296215   .0475938    -7.69   0.000     .2483769    .4374413
                                     -------------+--------------------------------------------------------------
                                          /lnsig2u |   .6331254   .2923795                       .0600722    1.206179
                                     -------------+--------------------------------------------------------------
                                           sigma_u |   1.372402   .2006311                       1.030492    1.827757
                                               rho |   .3640747    .067693                       .2440181    .5038327
                                     ----------------------------------------------------------------------------
Note: Estimates are transformed only in the first equation to odds ratios.
Note: _cons estimates baseline odds (conditional on zero random effects).
LR test of rho=0: chibar2(01) = 28.97                 Prob >= chibar2 = 0.000
```

Hausman test

```
                                                        ---- Coefficients ----
                                                  |      (b)          (B)            (b-B)     sqrt(diag(V_b-V_B))
                                                  |       fe           re         Difference       Std. err.
                                     -------------+--------------------------------------------------------------
            Knows the existence of the new policy |    .9582382     1.339131       -.3808928        .2073605
                                Year (ref. = 2019) |
                                             2021  |   -.4567584    -.7080786        .2513202        .1426987
                                     ----------------------------------------------------------------------------
                                        b = Consistent under H0 and Ha; obtained from xtlogit.
                         B = Inconsistent under Ha, efficient under H0; obtained from xtlogit.

Test of H0: Difference in coefficients not systematic

    chi2(2) = (b-B)'[(V_b-V_B)^(-1)](b-B)
            =   3.42
```

```
Prob > chi2 = 0.1809
```
